# To differentiate benign from malignant thyroid nodule comparison of sonography with FNAC findings

**DOI:** 10.12669/pjms.291.2595

**Published:** 2013

**Authors:** Mehrali Rahimi, Nazanin Farshchian, Eilkhan Rezaee, Karon Shahebrahimi, Hamid Madani

**Affiliations:** 1 Mehrali Rahimi, Associate Professor, Department of Endocrinology, Medical School, Kermanshah University of Medical Sciences, Kermanshah, Iran.; 2 Nazanin Farshchian, Assistant Professor, Department of Radiology, Medical School, Kermanshah University of Medical Sciences, Kermanshah, Iran.; 3 Eilkhan Rezaee, Researcher, Department of Radiology, Medical School, Kermanshah University of Medical Sciences, Kermanshah, Iran.; 4 Karon Shahebrahimi, Assistant Professor, Department of Endocrinology, Medical School, Kermanshah University of Medical Sciences, Kermanshah, Iran.; 5 Hamid Madani, Associate Professor, Department of Pathology, Medical School, Kermanshah University of Medical Sciences, Kermanshah, Iran.

**Keywords:** Thyroid nodules, Fine-Needle Aspiration (FNA), Sonography, Malignancy

## Abstract

***Objectives:*** To evaluate the diagnostic accuracy of sonography and Fine Needle Aspiration Cytology (FNAC).

***Methodology:*** This follow-up study was approved by review board and conducted at Endocrine Clinic and Radiology Department of Imam Reza, Kermanshah. The patients were diagnosed to have thyroid nodule examined by FNA and Sonography suspicious malignant cases underwent surgery. Results were entered in SPSS 11.5 chi-Square and Fisher exact test applied to compare malignant and benign nodule characters.

***Results:*** In this study 144 patients were examined and 14 cases (9.7%) had malignant nodule. Most of malignant nodules were single (p=0.001), solid (p < 0.001), hypo-echo (p=0.001), with irregular margins (p < 0.001) and with calcification (p=0.041). There was no significant relationship between malignancy and nodule size of larger than 15 mm (p=0.395). Compared with surgery, FNA sensitivity and specificity were calculated as 92.8% and 100% respectively.

***Conclusion:*** Based on the result of this study, thyroid nodule size must not be considered as a criterion for malignancy and thyroid nodules of any size must be suspected as malignant. Important criteria for malignancy include irregular edges, being solid, hypoechogenicity and being a single nodule respectively. Compared with Surgery, FNA Sensitivity and specificity were calculated as 92.8% and 100% respectively.

## Introduction

 Thyroid nodule is a common clinical finding and it is found in the autopsy of approximately 50% of individuals.^1^ Based on clinical examination, its prevalence is about four to seven percent.^2^^,^^3^ In the past two decades, the widespread use of ultrasonography for evaluation of thyroid and non-thyroid neck diseases has increased the prevalence of thyroid nodes; the prevalence rate has varied among different studies.^4^^-^^6^ Because of ruling out malignancy, thyroid nodules are clinically important and their role is proved for approximately five percent of nodes.^7^

 Most thyroid nodules (about 67%) have a size smaller than 15 mm.^8^ Although previously it was assumed that the nodes smaller than 15 mm are not malignant and do not need further investigations^5^, recently a number of studies have rejected this assumption and have shown small nodules can also be malignant and must therefore be investigated further.^8^^,^^9^ It is widely accepted that Sonography and ultrasound guided FNA Cytology are the modalities of choice for comparison of benign and malignant nodules.^10^^,^^11^ The diagnostic value of FNA in small nodules is still under study.^8^ However, if FNA is done on time when necessary, it can prevent unnecessary surgeries.^12^ Therefore, in these cases it can be valuable to be familiar with malignancy features in FNA and sonography.

 The definite diagnosis of malignancy is not possible through sonography, however some specifications may help finding suspected malignant thyroid nodules; nevertheless these specifications may exist in both types of nodes.^8^^,^^13^^,^^14^ Several studies have been conducted to assess the diagnostic value of sonography in differentiating benign from malignant nodes and the results have been different. Some finding such as echogenicity, the internal echotexture of thyroid nodes, nodes edge, calcification and its type, and the presence or absence of Halo has failed to differentiate malignant or benign nodes decisively and Fine-Needle Aspiration is recommended in all cases.^8^^,^^13^^-^^15^

 The aim of this study was to compare sonographic findings in benign and malignant thyroid nodules.

## Methodology

 This follow-up study was approved by the ethics committee of Kermanshah University of Medical Sciences. One hundred forty four patients with thyroid nodules who were referred to endocrine clinic of Taleghani or Imam Reza Hospital in Kermanshah in the time period of 2010-2011 were enrolled in the study.

**Table-I:** Comparing malignant and benign nodules based on personal and sonographic features.

*Individual or group features*	*Benign (Sum=130)*	*malignant* *(Sum=14)*	*Odd ratios (confidence interval of 95%)*	*The significance level*
Gender				
Female	127 (97.7%)	14 (100%)	-	1†
Male	3 (2.3%)	0 (0%)		
Number of nodules			5.89 (1.87- 18.55)	
Single nodule	24 (18.5%)	8 (57.1%)		0.003*
Multi nodule	106 (81.5%)	6 (42.9%)		
Nodule type			22.96 (2.91-181)	
Solid	47 (36.2%)	13 (92.9%)		<0.001†*
Cystic and Soft	83 (63.8%)	1 (7.1%)		
echogenicity Status			8.44 (1.81-30.27)	
Hypoechoechogenicity	54 (41.5%)	12 (85.7%)		0.003†*
Hyperechoic*/*isoechoic	76 (58.5%)	2 (14.3%)		
Nodule edge status			23.62 (5.52-101)	
irregular	4 (3.1%)	6 (42.9%)		<0.001†*
Regular	126 (96.9%)	8 (57.1%)		
Halo			3.87 (0.83-18.03)	
Without halo	79 (60.8%)	12 (85.7%)		0.083†
With halo	51 (39.2%)	2 (14.3%)		
Nodule size				
Larger than 15 mm	72 (55.4%)	10 (71.4%)		0.395†
Smaller than 15 mm	58 (44.6%)	4 (28.6%)	2.01 (0.6-6.75)	
Calcification				
With calcification	25 (19.2%)	6 (42.9%)		0.041*
Without calcification	105 (80.8%)	8 (57.1%)	3.15 (1-9.9)	

†Fisher test was used for comparison.

* The difference was statistically significant.

 Ultra Sound guided FNAC performed up to 3 passes per nodules can be made with the help of 21-22 gauge needles and 5 or 10cc syringes for procedure. Confirmed placement of the needle in targeted nodule can be achieved under U/S guidance. Try to take sample from solid portion of nodule if its complex nodule specimen prepare on glass slides and fix in 95% ethanol and send for cytology. A radiologist performs thyroid sonography for the patients using a GE ultrasound machine, P5 model, with a 10 MHZ linear probe. Radiology specialist recorded ultrasound characteristics such as size, internal structure, echogenicity, the edge status, presence or absence of Halo and calcification. If the FNA result was suspicious of malignancy, surgery was performed and pathology results were recorded. Exclusion criteria included receiving radioactive iodine or any history of thyroid surgery. Results were entered in SPSS 11.5 and chi-square and Fisher tests were used to compare malignant and benign nodules.

## Results

 In this study 144 patients were examined; 141 patients (97.9%) were females and the others were males. Their mean age was 39.8 (±12) years. None of the patients had a history of neck irradiation in childhood. Only one of the patients with benign nodule had the history of papillary carcinoma among family members (sister of the patient). From all nodules, 32 (22.2%) were single and 112 (77.8%) were multiple nodules; 67 nodules (46.5%) were solid and cystic (Mix). Concerning echogenicity, 66 nodules (45.8%) were Hypo-echo. 134 nodules (93.1%) had a regular edge. 91 nodules (63.1%) had no Halo. 82 nodules (56.9%) were larger than 15mm. 

 After surgery and pathology, 14 cases (9.7%) were reported malignant while 13 cases (9%) were confirmed malignant in FNA. All of these nodes were papillary thyroid carcinoma.

 There was no significant relationship between gender and malignancy (p=1). Most of malignant nodules were single nodules (p=0.001) and solid (p<0.001). There was a significant relationship between the mass hypo-echoism and malignancy (p=0.003). Most malignancies had irregular edges (p<0.001) and calcifications (p=0.041). There was no significant relationship between malignancy and nodule size of larger than 15mm (p=0.395). Compared with surgery, FNA sensitivity and specificity were calculated as 92.8% and 100%, respectively. Kappa coefficient was calculated as 0.959.

## Discussion

 In this study the prevalence of malignant nodules was 9.7 percent. Compared with surgery, FNA sensitivity and specificity for diagnosis of nodules were 92.8% and 100%, respectively and Kappa coefficient was calculated as 0.959. Being a single nodule, being solid, being hypo-echo, having irregular edges or calcification were the appropriate characteristics for differentiating malignant from benign nodules while the nodule size did not have appropriate differential value.

 In other studies, the prevalence of malignancy has been different. From all, 3.6% to 9.9% of all thyroid nodules have been reported malignant.^14^^,^^16^^-^^18^ In our study the prevalence of malignancy was about the same. In most studies, age and sex were not associated with malignancy.^6,9,19^ In addition in most studies the sensitivity and specificity of FNA have been better than surgery; hence using FNA together with sonography can be very efficient even for small nodules.^6^^,^^20^ FNA had high sensitivity and specificity in our study.

 Some studies have been conducted to assess sonography parameters in differentiating malignant from benign thyroid nodules; the results have been inconsistent, and it is still controversial.^14^^,^^21^ In a study in US, sonographic features failed to differentiate benign and malignant thyroid nodules and fine-needle aspiration was recommended for all cases.^14^ In some studies sonography had been unable to differentiate malignant and benign cases and FNA is recommended for all thyroid nodules regardless palpability.^15^^,^^22^ In a study, none of sonography characteristics, except calcification, was able to differentiate benign and malignant thyroid nodes.^14^

However, there are studies in favor of the usefulness of sonography markers in differentiating malignant from benign nodules. In a study, having a single nodule, irregular edges, and micro-calcification increased the chance of malignancy 3.6, 5.4 and 39 times, respectively.^6^ In Taneri et al study^23^, having multi nodules was associated with malignancy, while in Ugurlu et al study^6^ having a single nodule or two nodules increased the chance of malignancy and in Cappelli et al study^16^ being solid and hypo-echo were associated with malignancy. However in another study hypoechoechogenicity was not associated with malignancy.^6^ Unclear edges, irregular shape, being solid and hypoechoechogenicity can increase the chance of malignancy.^8^^,^^21^^,^^24^ In another study, a greater percentage of malignant nodules had irregular edges and hypoechoechogenicity.^22^ In Moon et al study^25^ irregular shape was not associated with malignancy but there was higher percentage of hypoechoechogenicity in malignant nodes. Some studies were in favor of sonography markers for differentiating malignant and benign cases, however none of them can prove the malignancy decisively. 

 Our study showed that the smallness of nodule cannot eliminate the chance of malignancy and it is required for all nodules of any size to be investigated further. As mentioned in other studies, there is no difference regarding malignancy between nodules smaller or larger than 10 mm.^26^ Cappelli et al^16^ study showed that considering thyroid tumors of larger than 10mm resulted in not detecting 19% of malignancies. Other studies have also questioned using exact sizes for suspecting malignant nodules.^9^^,^^14^ In a study it is recommended to do FNA even for 5mm nodules.^15^ In another study, nodes larger than 10mm did not increase the chance of malignancy.^6^ Therefore, it seems that the thyroid nodule size is not a good indicator for future actions, such as FNA or surgery, and malignancy must be suspected in nodules of any size.

 Our study also had limitations. One of its limitations was the small sample size; therefore it was not possible to use logistic regression analysis. It is recommended to conduct a similar study with larger sample size in order to identify the malignancy markers more accurately. A meta-analysis study is also recommended.

## Conclusions

 Based on the result of this study, thyroid nodule size must not be considered as a criterion for malignancy and thyroid nodules of any size must be suspected as malignant. Important criteria for malignancy include irregular edges, being Solid hypoechogenicity and being a single nodule respectively. Compared with Surgery, FNA Sensitivity and specificity were calculated as 92.8% and 100% respectively.
